# Significance of mental health legislation for successful primary care for mental health and community mental health services: A review

**DOI:** 10.4102/phcfm.v10i1.1429

**Published:** 2018-03-29

**Authors:** Getinet Ayano

**Affiliations:** 1 Research and Training Department, Amanuel Mental Specialized Hospital, Addis Ababa, Ethiopia

## Abstract

**Background:**

Mental health legislation (MHL) is required to ensure a regulatory framework for mental health services and other providers of treatment and care, and to ensure that the public and people with a mental illness are afforded protection from the often-devastating consequences of mental illness.

**Aims:**

To provide an overview of evidence on the significance of MHL for successful primary care for mental health and community mental health services

**Method:**

A qualitative review of the literature on the significance of MHL for successful primary care for mental health and community mental health services was conducted.

**Results:**

In many countries, especially in those who have no MHL, people do not have access to basic mental health care and treatment they require. One of the major aims of MHL is that all people with mental disorders should be provided with treatment based on the integration of mental health care services into the primary healthcare (PHC). In addition, MHL plays a crucial role in community integration of persons with mental disorders, the provision of care of high quality, the improvement of access to care at community level. Community-based mental health care further improves access to mental healthcare within the city, to have better health and mental health outcomes, and better quality of life, increase acceptability, reduce associated social stigma and human rights abuse, prevent chronicity and physical health comorbidity will likely to be detected early and managed.

**Conclusion:**

Mental health legislation plays a crucial role in community integration of persons with mental disorders, integration of mental health at primary health care, the provision of care of high quality and the improvement of access to care at community level. It is vital and essential to have MHL for every country.

## Background

About 14% of the global burden of disease is explained by mental disorders; mostly chronically disabling illness, depression and other common mental disorders such as psychosis^1,2^ and this will rise to 15% by the year 2020. For disability alone, without the effects of premature mortality, the impact of neuropsychiatric conditions is starker still: they account for 31% of all years lived with disability. The stigma and violations of human rights directed towards people with these disorders compound the problem.^3^

All people with mental disorders have the right to receive high-quality treatment and care delivered through responsive health care services. They should be protected against any form of inhuman treatment and discrimination.^4^

Mental health legislation (MHL) is required to ensure a regulatory framework for mental health services and other providers of treatment and care, and to ensure that the public and people with a mental illness are afforded protection from the often devastating consequences of mental illness.^4,5^

In many countries, especially in those that have no MHLs, people do not have access to basic mental health care and treatment they require. In others, the absence of community-based mental health care means the only care available is in psychiatric institutions, which are associated with gross human rights violations including inhuman and degrading treatment and living conditions. Even outside the health care context, they are excluded from community life and denied basic rights such as shelter, food and clothing, and are discriminated against in the fields of employment, education and housing because of their mental disability. Many are denied the right to vote, marry and have children. As a consequence, many people with mental disabilities are living in extreme poverty, which in turn affects their ability to gain access to appropriate care, integrate into society and recover from their illness.^4,5^

Scientific evidences have showed that neuropsychiatric disorders account for 13% of the global burden of disease and more than 75% of this burden was found in the low-and middle-income countries (LMICs).^4,5,6,7^ Results from different studies have shown that only a minority of people with mental disorders receive treatment, and even fewer receive high-quality treatment from mental health experts in the LMICs.^8,9^ Studies showed that between 76% and 84% of individuals with serious mental illness(SMI) did not receive treatment for their mental health disorders, representing a very high treatment gap.^8,9^ The World Health Organization (WHO) declared that to reduce the global mental health treatment gap, a possible solution is to integrate mental healthcare services into the primary healthcare (PHC) centres. For this reason, the WHO introduced the Mental Health Gap Action Programme, with the specific aim of scaling up services for mental, substance use and neurological disorders.^10^

Evidence from WHO has shown that only about one-third(36%) of the people living in low-income countries are covered by MHL, whereas the corresponding rate of coverage in most of the high-income countries is 92%.^3^ These data support the finding evidences indicating low access to mental health services because of the absence of MHL, that is, in low-income countries, mental disorders that are not considered as life-threatening problems are not given attention for a long time.^11,12^ As a result, mental health services are not given due priority and the needs of people for mental health care are not met.^13,14^ Untreated mental disorders lead to disability, substantial personal burden for affected individuals and their families, poor quality of life, human rights abuses, stigma and discrimination, poverty, decreased productivity, suffering, poor physical health and premature mortality.^15,16,17,18,19^

The presence of MHLs helps people with a mental disorder to get the best possible care and treatment appropriate to their needs, in the least restrictive environment and in the least intrusive manner consistent with the effective delivery of that care and treatment.^4^ According to basic and key standards of MGLs, all people with mental disorders should be provided with treatment based in the community except in very rare circumstances, that is, if there is a risk of self-harm or harm to other people or if the treatment can only be provided in an institutional setting.^4^ Community mental health (CMH) service is a treatment philosophy based on the social model of psychiatric care that advocates that a comprehensive range of mental health services be readily accessible to all members of the community. Integration into community-based rehabilitation (CBR), integration into PHC services and specialist CMH programmes are the common models of successful community-based mental health services.

## Methods

The aim of this study is to provide an overview of evidence in order to inform potential policy makers and direct researchers that the presence of MHLs plays a crucial role in community integration of persons with mental disorders, integration of mental health at primary health care, the provision of care of high quality and the improvement of access to care at community level.

Given the breadth of literature relating to both CMH and primary mental health care services, and how MHL plays a significant role for successful primary care in mental health and CMH services, it was decided to initially identify relevant review articles within the recent literature, and then use these to identify other key empirical pieces. The author searched the Cumulative Index of Nursing and Allied Health Literature (CinAHL), Excerpta Medica Database (Embase), Medlars Online (Medline), Psychological literature (PsycINFO) and the Cochrane Library for relevant studies. In order to facilitate subsequent identification of yet unknown key issues within the literature, selection criteria were purposefully broad. Papers whose central focus was not primary mental health care, CMH services and MHL were excluded.

## Results and discussion

To my knowledge, there are no such reviews aimed at relating significance of MHL for successful primary care for mental health and CMH services.

The literature demonstrated that the presence of MHLs plays a crucial role in community integration of persons with mental disorders, integration of mental health at primary health care, the provision of care of high quality and the improvement of access to care at community level. The details are discussed below.

## Mental health legislations and primary care for mental health

One of the major important components and aims of MHLs is informing and enforcing that all people with mental disorders should be provided with treatment based on integration of mental healthcare services into the PHC except in very rare circumstances, that is, if there is a risk of self-harm or harm to other people or if the treatment can only be provided in specialised mental health care centres and in institutional settings. The presence of MHLs is important for integration of mental healthcare services for persons with mental disorders at primary health care level, the provision of care of high quality, the improvement of access to care and promoting mental health and preventing mental disorders.^4^

It is advisable to have MHLs especially in LMICs where specialised mental health care professionals are extremely scarce and integration of mental health services at primary health care level is important to address mental health care needs of communities. In the LMICs, most of the specialised mental health care professionals are concentrated in the large cities and, consequently, a treatment gap exists as a high proportion of people live in rural areas and therefore have no access to mental health services. As far back as the 1970s, the WHO recommended that psychiatry be firmly rooted in primary care in order to reduce the treatment gap for mental health disorders.^3^ In the LMICs, impediments to mental health care services in communities include the uneven distribution of mental health resources, problems of accessing services in remote locations and affordability and social acceptability in relation to ignorance and belief systems. Families often have to make out-of-pocket payments for these services because of non-availability of social support systems. Specifically, in the National Health Insurance Scheme (NHIS), there is limited coverage for mental health care. The resultant effect of all these impediments is the rising number of people with mental health disorders living on the streets, a major social problem requiring urgent attention. Integrating mental health services into primary care is the most viable way of ensuring that people receive the mental health care they need. People can access mental health services close to their homes, thus keeping families together and maintaining their daily activities, and also avoid indirect costs associated with seeking specialist care in distant locations. In addition, intervening at primary care level helps to minimise stigma and discrimination.^5^

In addition, MHLs play a significant role in integration of mental health in primary health care and to improve access to care. The integration will further improve access to mental healthcare within the city, increase acceptability, reduce associated social stigma and human rights abuse, prevent chronicity and physical health comorbidity will likely be detected early and managed.

## Mental health legislations and community-based mental health care

Community mental health services (CMHS) refers to a system of care in which the patient’s community, not a specific facility such as a hospital, is the primary provider of care for people with a mental illness. The goal of CMH services often includes much more than simply providing outpatient psychiatric treatment.^20^

Community integration of persons with mental disorders is the other major critical issue addressed by MHLs. The main goal of CMH services is to have a comprehensive range of mental health services be readily accessible to all members of the community. It is one of the core issues to be addressed by mental health law. According to the principle of MHLs (i.e. principle of the least restrictive alternative), all people with mental disorders should be provided with treatment based on CMH care services except in very rare circumstances, that is, if there is a risk of self-harm or harm to other people or if the treatment can only be provided in specialised mental health care centres and in institutional settings.

CMH services are more accessible and effective, lessen social exclusion and are likely to have fewer possibilities for the neglect and violations of human rights that were often encountered in mental hospitals. However, WHO notes that in many countries, the closing of mental hospitals has not been accompanied by the development of community services, leaving a service vacuum with far too many not receiving any care.^21^

CMH services are essentially specialised mental health services based in the community. They include day centres, CBR services, hospital diversion programmes, specialist CMH programmes, mobile crisis teams, therapeutic and residential supervised services, group homes, home help, assistance to families and other support services. Although only some countries will be able to provide the full range of community-based mental health services, a combination of components based on local needs and requirements is essential. In particular, strong CMH services are essential as part of any deinstitutionalisation programme, as well as to prevent unnecessary hospitalisation. People receiving good community care have been shown to have better health and mental health outcomes, and better quality of life, than those treated in psychiatric hospitals. To maximise effectiveness, strong links are needed with other services up and down the pyramid of care.

Community services include supported housing with full or partial supervision (including halfway houses), psychiatric wards of general hospitals (including partial hospitalisation), local primary care medical services, day centres or clubhouses, CMH centres and self-help groups for mental health.

The presence of MHLs plays a crucial role in community integration of persons with mental disorders, the provision of care of high quality and the improvement of access to care at community level. Community-based mental health care further improves access to mental healthcare within the city, leads to better physical and mental health outcomes and thus better quality of life, increases acceptability, reduces associated social stigma and human rights abuse, and prevents chronicity and physical health comorbidity through early detection and management.

## Conclusion

I found that only about one-third (36%) of people living in low-income countries are covered by MHL, whereas the corresponding rate of coverage in most high-income countries is 92%. In many countries, especially in those that have no MHLs people do not have access to basic mental health care and treatment they require. In others, the absence of community-based mental health care means the only care available is in psychiatric institutions, which are associated with gross human rights violations including inhuman and degrading treatment and living conditions.

The presence of MHLs plays a crucial role in community integration of persons with mental disorders, integration of mental health at primary health care, the provision of care of high quality and the improvement of access to care at community level. Community-based mental health care further improves access to mental healthcare within the city; leads to better physical and mental health outcomes, and better quality of life; increases acceptability; reduces associated social stigma and human rights abuse; prevents chronicity; and probably ensures early detection and management of physical health comorbidities. Therefore, it is advisable and crucial to have MHL for any country.
